# TIMP-1 and MMP-9 expressions in COPD patients complicated with spontaneous pneumothorax and their correlations with treatment outcomes

**DOI:** 10.12669/pjms.36.2.1244

**Published:** 2020

**Authors:** Hang Li, Kaihu Shi, Yang Zhao, Jin Du, Dinghui Hu, Zuntao Liu

**Affiliations:** 1 Hang Li, Department of Thoracic and Cardiovascular Surgery, Nanjing Integrated Traditional Chinese and Western Medicine Hospital, Jiangsu Province, P. R. China; 2 Kaihu Shi, Department of Thoracic and Cardiovascular Surgery, Nanjing Integrated Traditional Chinese and Western Medicine Hospital, Jiangsu Province, P. R. China; 3 Yang Zhao, Department of Thoracic and Cardiovascular Surgery, Nanjing Integrated Traditional Chinese and Western Medicine Hospital, Jiangsu Province, P. R. China; 4 Jin Du, Department of Thoracic and Cardiovascular Surgery, Nanjing Integrated Traditional Chinese and Western Medicine Hospital, Jiangsu Province, P. R. China; 5 Dinghui Hu, Department of Thoracic and Cardiovascular Surgery, Nanjing Integrated Traditional Chinese and Western Medicine Hospital, Jiangsu Province, P. R. China; 6 Zuntao Liu, Department of Thoracic and Cardiovascular Surgery, Nanjing Integrated Traditional Chinese and Western Medicine Hospital, Jiangsu Province, P. R. China

**Keywords:** Chronic obstructive pulmonary disease, Matrix metalloproteinase-9, Tissue inhibitor of metalloproteinase-1

## Abstract

**Objective:**

To study the expressions of TIMP-1 and MMP-9 in patients with chronic obstructive pulmonary disease (COPD) complicated with spontaneous pneumothorax, and their correlations with treatment outcomes.

**Methods:**

A total of 80 COPD patients complicated with spontaneous pneumothorax treated in our hospital from December 2015 to December 2017. The serum expressions of TIMP-1 and MMP-9 in 80 COPD patients complicated with spontaneous pneumothorax (COPD group) and 52 healthy volunteers (control group) were detected by ELISA. The correlations of TIMP-1 and MMP-9 expressions with arterial blood gas parameters as well as scores of MRC breathlessness scale and St. George’s Respiratory Questionnaire (SGRQ) were analyzed.

**Results:**

The serum expressions of TIMP-1 and MMP-9 of COPD group were significantly higher than those of control group (P<0.05), but the two groups had similar MMP-9/TIMP-1 ratios (P>0.05). For COPD group, TIMP-1 expression, MMP-9 expression, MMP-9/TIMP-1, Sa(O_2_) and p(O_2_) were not correlated (P>0.05). TIMP-1 expression was significantly positively correlated with MRC scale and SGRQ scores (P<0.05). Sa(O_2_), p(O_2_) and MRC scale score of low MMP-9 expression, low TIMP-1 expression and low MMP-9/TIMP-1 group were significantly improved compared with those of high MMP-9 expression, high TIMP-1 expression and high MMP-9/TIMP-1 group (P<0.05). MMP-9 expression, TIMP-1 expression or MMP-9/TIMP-1 was not correlated with improvement of SGRQ score. Pulmonary function improvement (Sa(O_2_) improvement rate ≥5% and/or p(O_2_) improvement rate ≥10%) was correlated with serum MMP-9 expression, baseline Sa(O_2_) and p(O_2_).

**Conclusion:**

Increase of serum TIMP-1 and MMP-9 expressions in COPD patients was correlated with symptoms and scores of quality of life, and the expressions were also correlated with short-term treatment reactivity.

## INTRODUCTION

Chronic obstructive pulmonary disease (COPD) is a common clinical disease typified by airflow limitation that is incompletely reversible and evolves progressively, being closely related with abnormal inflammatory responses of the lung.^1^ Due to high morbidity rate, disabling rate and mortality rate as well as long disease course, COPD has attracted widespread attention recently.^2^-^4^ On the other hand, pneumothorax may occur owing to repeated pulmonary infection and chronic pulmonary fibrosis that damage alveolar structures and lead to their rupture at moderate mechanical pressure. As a common pleural disease, spontaneous pneumothorax occurs even without traumas or anthropogenic factors, during which lung tissue and visceral pleura spontaneously rupture owing to lung diseases or lung bullae and small emphysematous lesions near the lung surface rupture. As a result, the air in the lung and bronchus enters the pleural cavity, which then affects cardiopulmonary functions, mostly in the case of COPD. When complicated with spontaneous pneumothorax, COPD further jeopardizes pulmonary function, endangering patient health by inducing severe ischemia as well as cardiopulmonary function failure.

The symptoms and manifestations of COPD include chronic inflammations of the airway, pulmonary vessels and parenchyma, together with the resulting repeated injury and repair of the airway wall and airway remodeling.^5^ In recent years, the balance between proteases and antiproteases has been verified as a main factor to prevent damages to normal lung tissue structures. The degradation and deposition of extracellular matrix (ECM) lead to structural abnormalities of the airway wall, and serum matrix metalloproteinase-9 (MMP-9) primarily regulates the degradation and synthesis of ECM, also being able to degrade the matrix membrane. It also participates in inflammatory response and tissue reconstruction by mediating inflammatory cell accumulation and damaging epithelial/endothelial structures. As a result, incompletely reversible airflow limitation takes place.^6^,^7^ Tissue inhibitors of metalloproteinases (TIMPs) are a group of low-molecular-weight glycoproteins that can suppress the activities of MMPs. They are synthesized and secreted by macrophages, endothelial cells, fibroblasts and tumor cells, and then secreted into ECM as soluble forms. TIMPs inhibit the activities of MMPs by blocking their zymogen self-activation and specifically binding zinc ion in the catalytically active center to form stable complexes. For instance, TIMP-1 can bind the carboxyl terminal of catalytic center in MMP-9 to form an enzyme-inhibitor complex through noncovalent bonding, thereby specifically suppressing the activity of MMP-9.^8^ Thereby motivated, we herein studied the serum expressions of TIMP-1 and MMP-9 in COPD patients complicated with spontaneous pneumothorax, and their correlations with score of MRC breathlessness scale and score of St. George’s Respiratory Questionnaire (SGRQ).

## METHODS

### Baseline clinical data

This study has been approved by the ethics committee of our hospital, and written consent has been obtained from all patients. A total of 80 COPD patients complicated with spontaneous pneumothorax treated in our hospital from December 2015 to December 2017 were selected as a COPD group, including 35 males and 45 females aged 50-87 years old, (66.7 ± 8.2) on average. Inclusion criteria: In accordance with the diagnostic criteria for COPD in “Guidelines for Diagnosis and Treatment of COPD”; complication with spontaneous pneumothorax confirmed by chest CT and X-ray examination.^9^ Meanwhile, 52 healthy volunteers were selected as a control group, including 23 males and 29 females. Their baseline clinical data matched well with those of the COPD group, without respiratory tract symptoms, heart diseases, respiratory dysfunction, or history of respiratory tract infections four weeks before admission. The two groups had similar ages and gender ratios (P>0.05).

### Methods

After admission, pulmonary function as well as MRC scale and SGRQ scores were assessed, and venous blood was collected to detect serum MMP-9 and TIMP-1 expressions. Afterwards, the COPD group was subdivided into a high expression (H) group and a low expression (L) group according to the averages of MMP-9 expression, TIMP-1 expression and MMP9/TIMP1 ratio (319.51 ng/mL, 496.72 ng/mL and 0.643 respectively). The patients with lung tissue compression of <30% were subjected to oxygen therapy and airway spasmolysis, and the air was exhausted using closed drainage catheter if their symptoms were not relieved. For the patients with lung tissue compression of >30%, the air was exhausted using drainage catheter. Thoracoscopic surgery was conducted only when air leakage persisted for 1-2 weeks and the patients had good health status. After surgery, antibiotics were routinely administered to prevent infections of the respiratory tract, and hormones and cardiotonics were used to prevent pulmonary edema and to protect the myocardium. The above data were tested again three weeks later to compare the treatment outcomes.

### Pulmonary function detection

Arterial blood gas parameters, including Sa(O_2_) and p(O_2_), were detected with an arterial blood gas analyzer. The degree of breathlessness was evaluated by MRC scale,^10^ and health-related quality of life was assessed with SGRQ. Serum MMP-9 and TIMP-1 expressions were detected by ELISA strictly according to the instructions of kit purchased from Bender MedSystems (Austria).

### Statistical analysis

All data were analyzed by SPSS 16.0. The categorical data were subjected to the t test, and those before and after treatment were compared with the paired t-test. Correlations between variables were analyzed by the Spearman’s correlation analysis. The pathological and physiological indices related with the improvement of arterial blood gas parameters, MRC scale score and SGRQ score were screened with logistic regression analysis.

## RESULTS

### Serum expressions of MMP-9 and TIMP-1

The serum expressions of MMP-9 and TIMP-1 of the COPD group were significantly higher than those of the control group (P<0.05), but the two groups had similar MMP-9/TIMP-1 ratios (P>0.05) (Table-I).

**Table-I:** Serum expressions of MMP-9 and TIMP-1.

Group	n	MMP-9 (ng/mL)	TIMP-1 (ng/mL)	MMP-9/TIMP-1
COPD	80	319.51±20.19	496.72±28.72	0.643±0.214
Control	52	254.17±19.92	421.58±20.12	0.604±0.209
t		18.263	16.419	1.032
P		<0.001	<0.001	0.304

COPD: Chronic obstructive pulmonary disease; MMP-9: matrix metalloproteinase-9;

TIMP-1: tissue inhibitor of metalloproteinase-1.

### Correlations of MMP-9 expression, TIMP-1 expression and MMP-9/TIMP-1 with arterial blood gas parameters, MRC scale score and SGRQ score

For the COPD group, TIMP-1 expression, MMP-9 expression, MMP-9/TIMP-1, Sa(O_2_) and p(O_2_) were not correlated (P>0.05). TIMP-1 expression was significantly positively correlated with MRC scale and SGRQ scores (r = 0.376 and 0.368 respectively, P<0.05) (Table-I and II).

**Table-II:** Arterial blood gas parameters, MRC scale and SGRQ scores of COPD group before and after treatment (x̄ ± SD, n=80).

	Before	After	t	P
Sa(O_2_) (%)	90.04±5.21	97.16±5.22	7.422	<0.001
p(O_2_) (mmHg)	64.33±5.33	73.52±5.32	10.915	<0.001
MRC scale score	2.50±0.41	1.60±0.39	12.560	<0.001
SGRQ score	52.63±3.52	36.42±3.23	26.693	<0.001

COPD: Chronic obstructive pulmonary disease; SGRQ: St. George’s Respiratory Questionnaire.

### Arterial blood gas parameters, MRC scale score and SGRQ score of COPD group

Sa(O_2_), p(O_2_), MRC scale score and SGRQ score of the COPD group were significantly improved after treatment compared with those before treatment (P<0.05).

### Arterial blood gas parameters, MRC scale score and SGRQ score of H and L groups

Sa(O_2_), p(O_2_) and MRC scale score of the L group were significantly improved compared with those of the H group (P<0.05). Nevertheless, they had similar SGRQ scores (P>0.05) (Table-III).

**Table-III:** Arterial blood gas parameters, MRC scale score and SGRQ score of H and L groups (x̄ ± SD)

	n		Sa(O_2_) (%)	p(O_2_) (mmHg)	MRC scale score	SGRQ score
H group	38	MMP-9 (ng/mL)	93.29±2.23	70.42±2.11	1.72±0.41	36.13±3.11
	35	TIMP-1 (ng/mL)	94.82±2.15	71.64±2.24	1.65±0.38	36.24±3.09
	42	MMP-9/TIMP-1	95.12±2.21	70.96±2.12	1.94±0.36	6.32±3.14
L group	42	MMP-9 (ng/mL)	99.28±1.23	76.19±1.45	1.395±0.23	36.78±3.72
	45	TIMP-1 (ng/mL)	98.36±1.22	74.86±1.37	1.32±0.18	36.56±3.74
	38	MMP-9/TIMP-1	98.15±1.21	75.48±1.18	1.15±0.22	36.56±3.32

MMP-9: Matrix metalloproteinase-9; SGRQ: St. George’s Respiratory Questionnaire;

TIMP-1: tissue inhibitor of metalloproteinase-1.

### Logistic regression analysis of variables related to improvement rates of Sa(O_2_) and p(O_2_)

Logistic regression analysis showed that pulmonary function improvement (Sa(O_2_) improvement rate ≥5% and/or p(O_2_) improvement rate ≥10%) was correlated with serum MMP-9 expression, baseline Sa(O_2_) and p(O_2_) (Table IV-VI).

**Table-IV:** Logistic regression analysis results of variables related to Sa(O_2_) improvement rate of ≥5%.

Variable	Regression coefficient	Standard deviation	Wald	P	Odds ratio	95%CI	

						Lower limit	Upper limit
Baseline Sa(O_2_) (%)	-3.112	1.221	6.476	0.009	0.041	0.003	0.487
Constant	4.205	1.411	8.797	0.002	67.076		

CI: Confidence interval.

**Table-V:** Logistic regression analysis results of variables related to p(O_2_) improvement rate of ≥10%.

Variable	Regression coefficient	Standard deviation	Wald	P	Odds ratio	95%CI	

						Lower limit	Upper limit
Baseline p(O_2_) (mmHg)	-2.601	1.161	4.798	0.024	0.076	0.005	0.743
Serum MMP-9 (ng/mL)	-0.009	0.003	4.582	0.033	1.001	0.974	0.997
Constant	7.648	3.112	6.121	0.013	2.100×10^3^		

CI: Confidence interval; MMP-9: matrix metalloproteinase-9.

**Table-VI:** Logistic regression analysis results of variables related to Sa(O_2_) improvement rate of ≥5% + p(O_2_) improvement rate of ≥10%.

Variable	Regression coefficient	Standard deviation	Wald	P	Odds ratio	95%CI	

						Lower limit	Upper limit
Baseline Sa(O_2_) (%) + p(O_2_) (mmHg)	-3.939	1.792	4.692	0.029	0.018	0.001	0.679
Constant	4.337	1.984	4.793	0.032	76.927		

CI: Confidence interval.

## DISCUSSION

MMPs can be divided into six categories according to its structural and functional characteristics. Among them, gelatinase A and B (MMP-2, MMP-9) have become research hotspots due to their wide range of substrates. MMP-9 is only expressed in a small amount under physiological conditions.^11^ During inflammatory stimulation or cell transformation, inflammatory cells such as alveolar macrophages and neutrophils are secreted as zymogens, and other lung structural cells such as fibroblasts and epithelial cells are also expressed and can be activated by a series of protease cascades.^12^,^13^ The natural inhibitors of MMPs, known as TIMPs, share a common cellular and tissue source with MMPs and specifically inhibits MMP activity.^14^ It has a cell growth factor-like effect that promotes fibroblast proliferation and collagen synthesis and causes extracellular matrix deposition and inhibition of its degradation. The dynamic balance between MMPs and TIMPs is the decisive factor in maintaining environmental stability within ECM.

Chronic inflammation is one of the most important pathogenesis of COPD. The persistence of chronic inflammation can lead to repeated airway wall damage and repair processes, and result in increased collagen content, scar tissue formation and structural remodeling, causing air stenosis, fixed airway obstruction and decreased elastic recoil of the lungs, which plays a crucial role in airflow limitation. In the process of airway inflammation damage remodeling, MMPs/TIMPs reflects the balance of the two processes. Under normal circumstances, MMP-9/TIMP-1 is in equilibrium; in the pathological state, if the ratio increases, it indicates that the airway wall is mainly inflammatory reaction; if the ratio decreases, it indicates that the airway wall is mainly repaired. It is suggested that the trend of fibrosis is accompanied by the increase of TIMP-1 to inhibit the action of MMP-9 and the degradation of ECM like collagen. The excessive increase of TIMP-1 relative to MMP-9 eventually leads to excessive deposition of extracellular matrix in the airway, which promotes the occurrence and development of airway remodeling.^15^ Exhaled MMP-9 and TIMP-1 were significantly increased in the stable phase of COPD, and the concentration in the acute exacerbation period was further increased and negatively correlated with arterial blood gas parameters.^16^

The results of this study showed that the expression of serum MMP-9 and TIMP-1 in patients with COPD combined with spontaneous pneumothorax was higher than that in the normal control group, but its expression was not correlated with arterial blood gas parameters such as Sa(O_2_) and p(O_2_), similar to the results of Engstrom et al.^17^ The reason after analysis may be because the COPD patients with spontaneous pneumothorax included in this study are in a stable period, and most of them are newly diagnosed patients, with relatively short course of disease, relatively light symptoms and fewer times of acute exacerbation. Therefore, it may be mainly manifested as local inflammation of the airways and relatively mild systemic inflammation, failing to show a correlation between typical biomarkers and arterial blood gas parameters. However, TIMP-1 was positively correlated with MRC scale and SGRQ total scores, suggesting that with the over-expression of serum TIMP-1, ECM is excessively deposited in the airway, so as to promote airway remodeling, reduction of arterial blood gas parameters, and shortness of breath and decrease of health-related quality of life.

This study confirmed that patients with COPD and spontaneous pneumothorax had improved their arterial blood gas parameters, MRC scale and SGRQ scores after treatment, suggesting effective treatment. The improvement of arterial blood gas parameters or shortness of breath was also correlated with MMP-9, TIMP-1 and their ratio before treatment. The lower the expression, the more obvious the improvement of arterial blood gas parameters or shortness of breath. The lower the expression of MMP-9, the less the degradation of ECM, the fewer pathological changes such as airway inflammatory reaction, subsequent fibrosis and airway remodeling. The expression of TIMP-1 is an indicator of airway fibrosis. The lower the expression, the less the deposition of ECM in the airway and the less the airway remodeling. Then the airflow limitation has a certain degree of reversibility. The COPD patients with spontaneous pneumothorax airway inflammation exudation, fibrosis and mild degree of remodeling have higher therapeutic responsiveness, which is also associated with the pathological basis of the disease. According to the results that a patient’s therapeutic response is related to the expression of MMP-9 and TIMP-1, the authors speculate that it is possible to reduce the expression of MMP-9 and TIMP-1 through early active intervention, and alleviate the severity of ECM degradation, airway inflammation, fibrosis and airway remodeling slow the decline of FEV1 in COPD patients, and improve the quality of life.

## CONCLUSION

In summary, the expression of MMP-9 and TIMP-1 in COPD patients with spontaneous pneumothorax was higher than that in normal controls, but the expression was not correlated with arterial blood gas parameters. The improvement of arterial blood gas parameters and symptoms after treatment had a certain correlation with MMP-9, TIMP-1 and their ratio. Therefore, MMP-9, TIMP-1 and their ratio may be a predictor of COPD treatment response.

### Authors’ Contribution:

**YZ, JD, DH & ZL** performed this study, analyzed clinical data and drafted this manuscript, responsible for integrity of research.

**HL & KS** designed this study and significantly revised this manuscript.
